# Who are the key players in a new translational research network?

**DOI:** 10.1186/1472-6963-13-338

**Published:** 2013-08-30

**Authors:** Janet C Long, Frances C Cunningham, Peter Carswell, Jeffrey Braithwaite

**Affiliations:** 1 Centre for Clinical Governance Research, Australian Institute of Health Innovation, University of New South Wales, Kensington 2052, Australia; 2 Centre for Primary Health Care Systems Research, Menzies School of Health Research, Charles Darwin University, Darwin 0815, Australia; 3 School of Population Health, University of Auckland, Auckland 1142, New Zealand

## Abstract

**Background:**

Professional networks are used increasingly in health care to bring together members from different sites and professions to work collaboratively. Key players within these networks are known to affect network function through their central or brokerage position and are therefore of interest to those who seek to optimise network efficiency. However, their identity may not be apparent. This study using social network analysis to ask: (1) Who are the key players of a new translational research network (TRN)? (2) Do they have characteristics in common? (3) Are they recognisable as powerful, influential or well connected individuals?

**Methods:**

TRN members were asked to complete an on-line, whole network survey which collected demographic information expected to be associated with key player roles, and social network questions about collaboration in current TRN projects. Three questions asked who they perceived as powerful, influential and well connected. Indegree and betweenness centrality values were used to determine key player status in the actual and perceived networks and tested for association with demographic and descriptive variables using chi square analyses.

**Results:**

Response rate for the online survey was 76.4% (52/68). The TRN director and manager were identified as key players along with six other members. Only two of nine variables were associated with actual key player status; none with perceived. The main finding was the mismatch between actual and perceived brokers. Members correctly identified two of the three central actors (the two mandated key roles director and manager) but there were only three correctly identified actual brokers among the 19 perceived brokers. Possible reasons for the mismatch include overlapping structures and weak knowledge of members.

**Conclusions:**

The importance of correctly identifying these key players is discussed in terms of network interventions to improve efficiency.

## Background

Key players can be crucial to the effective functioning of any network [1] and there is interest in looking at how we can support their role in improving network outcomes [2,3]. Key players are those people that are seen as well connected and well informed. They are opinion leaders or have power and influence. They have roles in coordinating effort and bridging gaps.

Professional networks are used increasingly in health care to organize groups of people across organisational and disciplinary boundaries, for example clinical networks that seek to develop new treatment and care pathways [4,5], chronic disease networks that bridge the gaps between hospital and community services and seek to integrate care [6,7], and translational research networks that drive collaborative interdisciplinary endeavours between universities and health care settings [8]. Despite their rise in use throughout health care, our understanding of the processes and structural features of such networks, and how they contribute to network outcomes is limited [4,9]. Our understanding of the role of key players in this is also limited especially in the health care setting as research has been largely undertaken in commercial and competitive settings [10,11]. A deeper understanding of the role of key players in collaborative health care settings is therefore essential. Evidence from such research can then be used to develop interventions to support beneficial key player functions and mitigate any costs of the role.

Networks have a flatter hierarchy than traditional health care structures such as hospitals, and negotiations between members are social interactions, built on trust. While we may intuitively understand key players and networks in general, social network analysis allows us to examine these social interactions empirically. Network analysis measures the relationships and interactions between members (actors) rather than their individual attributes. Networks are made up of nodes (the actors) and ties (the relationship links) between them and whole network and individual actor parameters can be calculated. The ties form the structure of the network and actors are said to hold positions within that structure [12]. Table 1 summarises social network terms used in this paper.

**Table 1:** **Social network terms**[13-17]

**Term**	**Definition**
Actor	Member of a network.
Broker	A go-between or bridge, linking two other actors that are not themselves linked.
Central actor	The actor who is nominated most often or who interacts with the most others in their network.
Indegree	The number of ties directed to a focal actor. E.g. if 5 people nominate Mary as a collaborative partner, Mary’s indegree is 5.
Network	A group of people having some form of interaction between them.
Node	Actors in a sociogram are depicted as nodes connected by ties.
Outdegree	The number of ties that an individual actor directs to other actors. E.g. if Mary nominates 3 people as her collaborative partners, Mary’s out degree is 3.
Sociogram	Graphical representation of the relationships between actors in a network.
Tie	Relationship between two actors depicted as a line in a sociogram. Ties must be clearly defined, as different relationships will give different patterns of ties (e.g. “who are your friends?” and “who do you report to?”).

Two network positions, the central actor and the broker, are considered key to network function and outcome [1,2]. The actor who is most central, with ties to the most other actors, has been shown to co-ordinate communication and effort across the network [13]. Central actors (or more correctly here, prestigious actors [18]) are often seen as experts or opinion leaders [19] and are the most sought after for advice within the network. These centrality characteristics make networks vulnerable to the loss of key players [1].

Brokers lie between actors who would not otherwise be linked, and facilitate transactions and the flow of information between them [10]. Within health care the presence of organisational silos and professional “tribes” mean that there are many such gaps between members [20-22]. Gaps between these unlinked agents may also be due to geographic position (for example members from different wards, hospitals or health regions), cultural or discipline specific differences (including seniority or status) or alternatively, reflect the fact that parties have little basis on which to trust others [10,23]. Brokers seem to be crucial to connecting marginalised or isolated members and for bridging boundaries imposed by hierarchy or perceived power [2]. Loss of a broker may result in the network fragmenting or compromising communication pathways [24]. Both central and brokerage roles have the potential to become onerous to the individual and may introduce workflow or communication bottlenecks which decrease network efficiency [25,26].

Formal network reporting and communication structures do not always match the informal or social structures that “actually get the work done” [27,28]. This has given rise to the concept of the “invisible network” [29,30]. Formal mandated roles and key player roles often overlap; the appointed network leader for example, is often the central actor. However, the role of other actors may be less visible. One may know exactly what is happening among one’s close and regular contacts (or strong ties) but it is more difficult to be aware of the ties among one’s less frequent contacts (or weaker ties) [28,31]. Therefore interventions that seek to support key players must first start by accurately identifying them and the network functions they influence [32].

We chose a translational research network (TRN) as a case study to explore the key player role in a health care setting. TRNs are designed to increase the focus of knowledge translation in health care [33,34], bringing together university-based researchers and hospital-based clinicians to develop new clinical practices and introduce them into routine patient care. Translational research is considered a priority in the research agenda of many countries for example the nine Collaboratives for Leadership in Applied Health Research & Care (CLAHRCs) in England and the large number of interorganisational alliances funded by the Clinical and Translation Science Awards in the US. Collaboration [35-38], innovation [39-41], knowledge exchange [36,42] and diffusion of findings [43-45] are all intended outcomes of TRNs. Key players have been associated with all these functions in a range of network settings [11,19,26,46-48]. It is these informal positions in a network that have been linked to increased idea generation and knowledge translation [49]. Gray [2] writing more generally about transdisciplinary networks hypothesized that brokers would be critical to bridge the gaps between the disciplines, while Haines and colleagues [50] see central actors as necessary for co-ordinating effort and disseminating findings in an academic transdisciplinary collaboration. So while these roles have been explored in other network settings they have yet to be examined within TRNs.

The importance for research to examine key players in TRNs was highlighted in a major international review of funding mechanisms designed to support the translation of research findings. This review highlighted the struggles to get linkages between agencies, researchers, and decision makers [51]. It implied that further research examining the network dynamics of these TRNs would help lead to better strategies to support the translation of evidence into practice. Another study looking at knowledge translation networks in the clinical domain reiterated this point, and highlighted the need for further research to focus on understanding the role knowledge brokers play in such networks [33].

In response to the need for advancing this area, this paper explores the important area of the informal positions in a network; [49] an area which can readily be addressed by social network analysis [52]. It highlights where the informal positions of influence and brokering are, if they are linked to any traditional characteristics of power (i.e. expert power), and if others recognise the informal network positions as having influence. Specifically, the paper asks three questions:

1. Who are the brokers and central actors in a network of current collaborators in a new TRN?

2. Do the key players have demographic and descriptive characteristics in common? For example, are the identified key players all clinicians, or senior academics, or have similar opinions about network barriers and their own role within the network?

3. Are the key players recognisable as powerful, influential or well connected individuals by other members of the TRN?

Addressing these questions will help provide guidance to funders of health based TRNs on how to structure and manage the networks, the sort of individuals that should be a part of the network, and the informal network positions that need to be identified and nurtured. This will go some way to fill the gaps in the knowledge base identified by international health services research examining TRNs [33,51].

## Methods

### Setting

The target TRN was initiated in late 2011 with a membership of 68 clinicians and researchers from 12 hospital and university campuses in New South Wales, Australia. The overarching goal of the network is “taking science to practice,” and is focused on putting clinically proven knowledge of disease processes, diagnostic or treatment techniques into routine clinical practice and health decision-making [53]. The network provides access to funding for short-term projects, shared databases and facilities, and support from project officers and translational research (TR) fellows. This study forms part of a larger project looking at network structure and key players [54].

The TRN is embedded in a pre-existing and complex inter-organisational network of long-standing research and teaching arrangements as well as self-initiated collaborative research. TRN activities and funded projects do not displace existing and ongoing research such as National Health and Medical Research Council (NH&MRC) funded projects. At its inception, the TRN was already a collaborative effort as core group members prepared and submitted the proposal to the funding body. Past collaborative ties and knowledge of a broad range of clinicians and researchers in the field therefore define the starting point of the network and give a base line for growth in linkages of members and new collaborations over time.

### Procedures

People listed as full members of the TRN as of January 2012 were asked to complete an on-line, whole network survey in March 2012, each member receiving a link to the secure survey site via personal email. Ethics approvals were obtained from the University of New South Wales and appropriate local health network and site-specific committees (Additional file 1). Respondents were assured of anonymity in the reporting of results (names being replaced by anonymous codes) and were required to give formal consent. We sought answers from all members to reflect the whole network, rather than a sample of members [14,55]. To maximise the response rate, three follow-up reminders were emailed to non-respondents over the succeeding two months.

The survey was divided into two sections (see Additional file 2). The first section collected demographic and descriptive information (not able to be sourced from TRN documents), which was expected to be associated with key player roles: main tasks, seniority, past and present places of work, previous involvement in translational research and membership in other relevant networks or communities of practice. As some members have a clinical role as well as a research role, it was anticipated that the concept of the “two cultures” of clinician or researcher [56] might not be clear-cut. Members were therefore asked to choose one title for themselves from clinician, manager, academic, researcher, clinical-researcher or “other.” We named this variable “Self Title.” Two survey questions asked about the importance to respondents of various network barriers and enablers identified in previous interviews as significant (5 item Likert scale). Other questions asked about reasons for joining the TRN and specific activities undertaken so far within the TRN. These questions were designed so we could categorise members as having either an altruistic or opportunistic approach to joining the network, and an active or passive attitude to their role in network activities. An altruistic approach where the actor is committed to the TRN and an active attitude to network activities were expected to be associated with central actor status particularly.

The second part of the survey asked social network relationship questions: the participant’s current links to each other member, as well as perceptions of who were key players. Each question asked about a different relationship or interaction and so produced four unique sociograms or network graphs: Current collaboration, Powerful, Influential and Well connected.

The Current Collaboration network graph was constructed from respondents’ answers to two specific questions: “Who are the people with whom you have consulted or collaborated regarding any TRN research project” and “Who are the people with whom you have collaborated regarding dissemination of TRN objectives or findings”.

Since centrality is often associated with network power and influence [10,57] and brokers are often thought of as well connected, go-betweens or bridges to other groups [24,29] we asked “who are the most powerful members (in terms of setting the agenda),” “who are the most influential members (in terms of achieving TRN outcomes), and “who are the most highly connected members?” Since the exact membership of the TRN was known, each question provided a roster of TRN members’ names, job titles and primary place of work as an aid to memory.

Key player parameters were calculated for each respondent in each of the four networks using UCInet v6 [58], a software tool for social network analysis. The parameters indegree and betweenness centrality were chosen to identify key players [13-15]. Indegree is a measure of how many other actors in the network nominated the actor of interest as a collaborator and is used here to determine the most central actor. Betweenness centrality measures the extent to which the actor of interest lies between other actors that are not themselves linked. It is an indication of brokerage potential.

Each member was categorised as being an identified key player in the actual (Current Collaboration) or perceived (Powerful, Influential or Well-connected) networks, or not a key player. Chi square analyses were used to test for any association with demographic variables of current workplace, gender, membership of other networks, more than one relevant qualification and survey answer patterns: altruistic approach, active role and barriers and enablers.

## Results

### General

The response rate for the online survey was 76.4% (52/68). Of these, three responses were incomplete while a further three members provided answers to the social network questions separately. Members named 16 different current workplaces. Since many workplaces had only one respondent, workplaces were aggregated into three broad sites for analysis (Central, Satellite and Peripheral sites), based partly on geographic proximity and partly on administrative proximity (largely NSW Local Health Districts). Sixty three per cent of respondents worked at a Central Site, 27.8% at a Satellite Site and 9.2% at a Peripheral Site. A comparison of respondents and non-respondents showed that they were similar in gender distribution (χ^2^ (1, n = 68) 1.85, p = 0.17) and representation from Central, Satellite and Peripheral Sites (χ^2^ (2, n = 68) 5.27, p = 0.072). Lowest response rates came from the Peripheral sites.

Most respondents (87.8%) chose a Self Title of “researcher”, “academic”, “clinician” or “clinician-researcher.” These categories were aggregated for analysis after consideration of respondent’s stated key tasks, workplace and qualifications. All who chose “academic” for example, had stated that research was their key role and were university-based so were classified “researchers.” This resulted in 38.8% with a Self Title of “clinician”, 53.7% “researcher” and 7.4% “clinician-researcher.” Members overwhelmingly considered themselves experts in their field (96.3%); 79.6% had over ten years experience in their field with 33.3% over 20 years.

Survey questions asking how important different factors were as barriers or facilitators of the network were explored using factor analysis (principal axis factoring with Varimax rotation). Three factors (with Eigenvalues exceeding 1.5) were identified as underlying the 19 survey items. In total, these factors accounted for around 49% of the variance in the data. Altruistic approach and Active role variables were generated from respondents’ answers. Table 2 contains a summary of variables tested for their relationship with key player status.

**Table 2:** Survey variables used to test for association with key player status

**Variable**	**Survey items**
Altruistic approach	Deeply committed to the TRN’s objectives, offering expertise, representing other workgroups, disseminating findings
Active role	Attended formal meetings of the TRN, initiated meetings, involved in a TRN project, provided advice for TRN projects
Resources *(Cronbach’s alpha: 0.76)*	Enabler: Adequate funding, access to shared resources and expertise, support of Project Officers and Fellows Barriers: Lack of adequate funding
No Incentives *(Cronbach’s alpha: 0.78)*	Barriers: Lack of incentives, lack of time, lack of interest from colleagues, difficulties of collaborations
Focus *(Cronbach’s alpha: 0.73)*	Enablers: Projects focused on patient outcomes, strong leadership, good communication, social capital of members Barriers: Poor or absent links between researchers and clinicians

**Figure 1:**
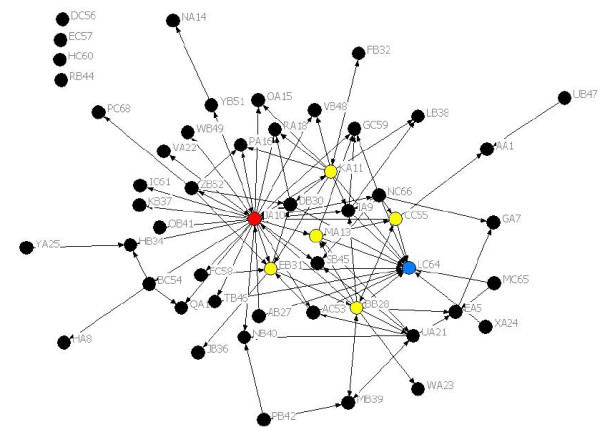
**Current Collaboration network.** Central actor LC64 shown in blue, brokers BB28, EB31, DB30, CC55, MA13 and KA11 in yellow and JA10 who is both a central actor and a broker is shown in red.

Figure 1 shows the Current Collaboration network diagram. The 52 respondents (49 for the Well-Connected question) gave answers for all 68 TRN members; i.e. we had outgoing ties for 52 and incoming ties for 68 members. We chose to use the matrix of ties just to and from the respondents after determining that the results were similar to those where we included and symmetrised incoming ties for all 68 members of the TRN (i.e. manually added an outgoing tie to every incoming tie to a non-respondent).

**Figure 2:**
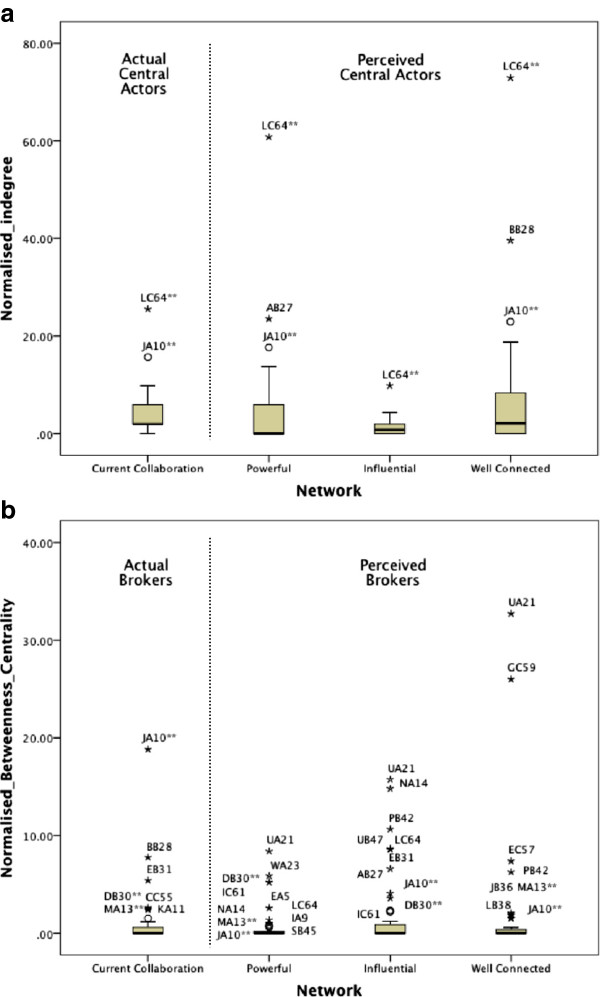
**Boxplots**^**1 **^**of key player values in each of the four networks: Current Collaboration, Powerful, Influential and Well Connected. (a)** Normalised indegree (outliers are top ranking central actors); **(b)** normalized betweenness centrality (outliers are considered the top ranking brokers). Actors marked with ** indicate they are both actual and perceived key players.^1^Boxplots show minimum, first quartile, median, third quartile, maximum and outliers shown with star or circle. The stars represent values greater than the third quartile plus three times the interquartile range while circles represent values greater than or equal to the third quartile plus 1.5 times the interquartile range.

Indegree and betweenness centrality values were computed for each actor in each network using UCInet [58] and as network size (N) varied between 49 and 52, normalised in regards to the network size (divided by 1/N and multiplied by 100). The range of values however were not normally distributed in any of the networks examined, each having a marked positive skew and between one and ten outliers. Since the distributions defied further standard methods of normalisation to allow comparisons (square root, natural log and reciprocal), regressions, ANOVAs and t-tests were ruled out. Instead, outlying actors with scores significantly above the mean were considered the top ranking key players in each network and key player status was used as a categorical variable with chi-square analyses to test for association with the various demographic and survey variables. Figure 2 shows boxplots of all four networks examined.

1. Who are identified as the key players in the Current Collaboration network?

Identified central actors (normalised indegree outliers) in the Current Collaboration network were LC64 (the TRN director), JA10 (the TRN manager) and EB31 with normalised indegree scores 25.5, 15.7 and 9.8 respectively. Mean indegree across all members of the network was 4.0. Identified brokers (betweenness centrality outliers) in the Current Collaboration network were: JA10, the TRN manager with a normalised betweenness centrality of 18.8, followed by Central Site researchers BB28, EB31, DB30 and KA11, then CC55, a clinician from a Satellite Site and MA13 a clinician from the Central Site. Mean betweenness centrality across all members of the network was 0.9.

2. Do key players have demographic variables or survey opinions in common?

Holding another relevant qualification and strongly agreeing with the importance of adequate resources for the TRN (Resources variable) were the only two variables to be significantly associated with key player status in the Current Collaboration network. Workplace and Self Title were not significantly associated with key player status. Table 3 summarises the full results of chi-square analyses.

3. Are the identified key players recognisable as powerful, influential or well connected individuals by the other members of the TRN?

**Table 3:** Chi square statistics for identified key players compared to the other TRN members by demographic and survey variables

**Identified key players**	**Variables**	**Chi square value**	**Degrees of freedom**	**Significance**
Actual (Current collaboration network)				
	Gender	0.42	1	0.517
	Workplace	2.06	2	0.358
	Member of another network	<0.01	1	0.971
	Other qualifications	6.02	1	0.014*
	Resources	5.37	1	0.020*
	No incentives	1.64	1	0.200
	Focus	1.40	1	0.238
	Altruistic approach	0.32	1	0.574
	Active role	0.66	1	0.418
Perceived (powerful, influential or well connected networks)				
	Gender	0.10	1	0.749
	Workplace	2.63	2	0.268
	Member of another network	0.86	1	0.354
	Other qualifications	<0.01	1	0.961
	Resources	3.00	1	0.083
	No incentives	2.54	1	0.096
	Focus	0.15	1	0.696
	Altruistic approach	<0.01	1	0.976
	Active role	2.19	1	0.139

*Indicates significance at α = 0.05.

Members accurately identified two of the three central actors in the Current Collaboration network but only three of the six brokers. Moreover, TRN members perceived two actors as central actors who were not and 16 actors as brokers who were not. There were far fewer actors perceived as central actors across the Powerful, Influential and Well-connected networks compared to those perceived as brokers (four compared to nineteen).

Respondents saw a difference between being powerful in terms of setting the agenda, and being influential in terms of achieving objectives. There are 16 perceived brokers across the Powerful and Influential networks but only five were identified in both networks. A further five actors were perceived as brokers in the Well connected network who were not identified as a broker or central actor elsewhere. Table 4 summarises the actual and perceived key players.

**Table 4:** Identified key players from the actual (Current Collaboration network) and perceived (“Who do you think are the most powerful or most influential or well-connected members of the TRN?”)

**ID**	**Gender**	**Self title**	**Work place**	**Role**	**Central actor**	**Perceived central actor**	**Broker**	**Perceived broker**
LC64	F	CR	Central	Director	CC	P, I, W		P, I
JA10	F	R	Periph	Manager	CC	P, W	CC	P, I, W
AB27	M	R	Satellite	Gov Body		P		I
BB28	M	R	Central	Gov Body		W	CC	
EB31	F	R	Central	Member	CC			I
DB30	M	R	Central	Member			CC	P, I
KA11	F	R	Central	Member			CC	
CC55	F	C	Satellite	Member			CC	
MA13	M	C	Central	Member			CC	P, W
UA21	M	CR	Central	Gov Body				P, I, W
WA23	M	R	Central	Gov Body				P
EA5	M	R	Central	Gov Body				P
IC61	F	R	Central	Member				P,I
SB45	F	R	Central	Member				P
NA14	F	C	Satellite	Member				P, I
IA9	M	C	Satellite	Member				P
PB42	F	C	Central	Member				I, W
UB47	F	C	Central	Member				I
GC59	F	C	Central	Member				W
EC57	F	C	Central	Member				W
JB36	M	R	Satellite	Member				W
LB38	M	C	Central	Gov Body				W

Key: *P* Powerful, *I* Influential and *W* Well-connected, *C* Clinician, *R* Researcher, *CR* Clinician-researcher.

## Discussion

This study found that the TRN director was by far the most central actor in the Current Collaboration network. This was not surprising as the director had positions across several sites and had been instrumental in inviting a large number of members to join the TRN. The TRN manager’s status as both a central actor and a broker was consistent with the mandated role as a facilitator and co-ordinator. The manager had not worked with any TRN members before joining the staff but had since worked assiduously to contact and facilitate connections between members. Factors associated with successful transdisciplinary teams include leadership that encourages collaboration, manages conflict between groups and clearly communicates team goals [59]. The director and manager’s centrality in the TRN places them in the best position to achieve this. There were six other members identified as key players, one central actor and five brokers. One of the brokers was also on the governing body, another mandated leadership position.

However, there was a lack of an association between key player status and most demographic and survey variables. For example, contrary to expectations, it was found that an active role in TRN activities (e.g. initiating a meeting to discuss a TRN project) was not associated with central actor status. The only significant association between key player status and the variables examined was for the survey item Resources and having another qualification relevant to the TRN. Central actor roles are often associated with expert status and so an extra qualification that implies a wider knowledge base and enhancement of expertise is consistent with this finding. The Resources survey item reflects the respondents’ strong agreement with the statement that access to the network’s resources such as shared databases, funding and Project Officer support are significant enablers of the network. All the identified key players in the Current Collaboration network strongly agreed whereas across the other respondents 24 strongly agreed and 18 agreed or were neutral. One of the stated roles of the TRN is to develop these resources and make them accessible to the members. The four key players in mandated positions would be especially aware of this and its importance. Moreover, since this is a configuration of relationships described by collaboration, it might be assumed that collaborators are using these new TRN resources and so may be especially appreciative of their importance.

The identified key players seem to have little else in common and this could be due to the historical context of the TRN. The pre-existing research and teaching relationships established before the TRN was formed (such as research grant collaborations or training arrangements through Clinical Schools) may have a greater influence on who is in a key player role than current involvement in TRN activities. Rather than considering the TRN to be a new network, for some members it may perhaps be perceived as just a reconfiguration of existing contacts.

The third aim of the study was to test whether members had accurate perceptions of who held key player positions in the network. This was the most significant finding of the study: the marked mismatch between actual and perceived brokers. For example, actor UA21, a clinician-researcher from the Central Site, is the top ranked broker in the Powerful and Well connected networks and ranks second in the Influential network but is not identified as an actual broker (or central actor) in the Current Collaboration network. All three perceived networks feature brokers that are not identified as key players in the Current Collaboration network. This implies that these members have a reputation of an active role and are well known in the pre-existing research or formal hospital networks but are not currently involved in TRN projects. In other words, key player activity in one setting may not necessarily carry across to another. While other explanations may be possible for this (e.g. UA21 may have been on leave during the first funding round) this does raise the issue of how the picture of who is “getting things done” [29] is further blurred when multiple networks having different member sets and purposes are overlaid as here: the formal university or hospital organisational structure, the network of past collaborative ties and now the TRN. Being aware of people’s roles in each of these separate network contexts may be difficult to tease out.

The rather simplistic popular conception of the brokerage role as a well connected individual is another factor which makes accurate identification of brokers difficult. While central actors are often fairly obvious as the person to whom most other members are in contact, the social network algorithm that computes brokerage potential measures the extent to which the member lies on the shortest path between unconnected members which is far more difficult to see, especially across a whole network.

Brokers have an important role to play in TRNs such as the network here [2]. The structure of this and many other TRNs is a hybrid of hierarchical and enclave structures [60]: hierarchical insofar as it has a central authority in the director and governing body. However, the general membership is intended to have a flat structure and egalitarian access to TRN resources. Brokers can be especially useful here by bridging and brokering power gaps that may persist from the more hierarchically structured hospital and university arenas [2]: bridging the gap between nurses and doctors or post graduate students and professors, linking more peripheral members (such as allied health clinicians or researchers based in rural sites) to the centrally located members.

If perceived key players are not necessarily the actual key players, there are implications for network management to consider. Planned interventions or role support may be targeted at the wrong people: an intervention aimed at supporting the brokerage role of UA21 in the Current Collaboration network would clearly be unsuccessful as UA21 is not in the correct position in this network to carry out the brokerage role. It may even compromise network resilience; since networks are particularly vulnerable to the loss of central actors and brokers, this risk can only be managed if the actual key players are identified.

How participants decided that other members were well connected is not apparent. Only three of the ten actors identified as key players in the Well connected network reported being a member of another relevant network or community of practice which was hypothesised to increase the perception that they are well connected.

It was expected that central actors would be considered experts in their field and hence sought out by others for collaborative engagement. However, the high number of experts and senior practitioners meant that this was not apparent. As the membership of the TRN grows and attracts more members that are less experienced, more junior and not currently engaged in translational research, expert status may become more closely linked with key player status.

### Strengths and limitations

Survey data collected for social network analyses rely on accurate self report. Every effort was made to ensure the accuracy of this data. Care was taken to make the relationship questions as unambiguous as possible (through incorporating feedback from earlier interviews and a pilot survey). This minimises the risk that reported ties are understood differently by respondents. Also a roster of members’ names and positions was used to provide every opportunity for a full list of collaborators to be reported.

There were, as reported earlier, a number of difficulties in analysing the relatively small data set, however the 76.4% response rate for the whole network was the best that a concerted follow-up effort directed at non-respondents could achieve. Aggregating categories decreased the power of analyses to show up patterns yet small frequencies necessitated this approach.

The survey was a snapshot and as such gives no information about the growth or efficiency of the network over time. Follow-up studies planned for a later date [54] will provide further data to examine changes over time and to link network structure and key player roles with network outcomes.

## Conclusion

Key players have positions of power and influence in networks and as such are of interest to people seeking to understand and optimize network function. Central actors are often seen as experts and most sought after for advice within the network while brokers can bridge or mediate connections between unlinked members. Any interventions to support key players must begin by correctly identifying them. This study has empirically identified these players. Most significantly, the perceptions about who is holding these influential positions are not always correct. We found that the TRN director was a central actor while the TRN manager was both a central actor and a broker. Both were correctly identified as such. However, identification of members in brokerage positions was far less accurate. Suggested reasons for this include: multiple overlying organizational structures that may blur what roles are done in what context, oversimplified ideas about what brokers do and the difficulty of knowing what happens among network members who lie outside one’s strong, close ties.

## Competing interests

JL is supported by a PhD Scholarship Top-up Award funded by the Cancer Institute NSW. No funding partners had a role in this research or papers published from it.

## Authors’ contributions

Study design and analysis was developed by all four authors. JL collected data, and wrote the paper, PC assisted with analysis, and FC, PC and JB critically reviewed drafts and the final copy. All authors read and approve the final manuscript.

## Pre-publication history

The pre-publication history for this paper can be accessed here:

http://www.biomedcentral.com/1472-6963/13/338/prepub

## Supplementary Material

Additional file 1 Ethics Approvals. Click here for file

Additional file 2 TRN Collaboration Survey 1 for BMC HSR. Click here for file

## References

[B1] Borgatti SP Identifying sets of key players in a social network Comput Math Organiz Theor 2006 12 21 10.1007/s10588-006-7084-x

[B2] Gray B Enhancing transdisciplinary research through collaborative leadership Am J Prev Med 2008 35 S124 S132 10.1016/j.amepre.2008.03.037 18619392 PMC2542584

[B3] Valente TW Network interventions Science 2012 337 49 53 10.1126/science.1217330 22767921

[B4] Cunningham F Ranmuthugala G Westbrook J Braithwaite J Net benefits: assessing the effectiveness of clinical networks in Australia through qualitative methods Implement Sci 2012 7 108 10.1186/1748-5908-7-108 23122000 PMC3541150

[B5] McInnes E Middleton S Gardner G Haines M Haertsch M Paul CL Castaldi P A qualitative study of stakeholder views of the conditions for and outcomes of successful clinical networks BMC Health Serv Res 2012 12 49 10.1186/1472-6963-12-49 22373078 PMC3325167

[B6] Greene A Pagliari C Cunningham S Donnan P Evans J Emslie-Smith A Morris A Guthrie B Do managed clinical networks improve quality of diabetes care? Evidence from a retrospective mixed methods evaluation Qual Saf Health Care 2009 18 456 461 10.1136/qshc.2007.023119 19955457

[B7] Fuller J Perkins D Parker S Holdsworth L Kelly B Roberts R Martinez L Fragar L Building effective service linkages in primary mental health care: a narrative review part 2 BMC Health Serv Res 2011 11 66 10.1186/1472-6963-11-66 21435273 PMC3070626

[B8] Sung NS Crowley WF Genel M Salber P Sandy L Sherwood LM Johnson SB Catanese V Tilson H Getz K Central challenges facing the national clinical research enterprise JAMA 2003 289 1278 1287 10.1001/jama.289.10.1278 12633190

[B9] Braithwaite J Westbrook JI Ranmuthugala G Cunningham F Plumb J Wiley J Ball D Huckson S Hughes C Johnston B The development, design, testing, refinement, simulation and application of an evaluation framework for communities of practice and social-professional networks BMC Health Serv Res 2009 9 162 10.1186/1472-6963-9-162 19754942 PMC2751758

[B10] Burt RS Structural holes: the social structure of competition 1992 Cambridge, Massachusetts: Harvard University Press

[B11] Long J Cunningham FC Braithwaite J Bridges, brokers and boundary spanners in social professional networks: a systematic review BMC Health Serv Res 2013 13 158 10.1186/1472-6963-13-158 23631517 PMC3648408

[B12] Borgatti SP Halgin D On network theory Organization Science 2011 22 1168 1181 10.1287/orsc.1100.0641

[B13] Freeman LC Centrality in social networks: conceptual clarification Social Networks 1979 1 215 239

[B14] Wasserman S Faust K Social network analysis 1994 Cambridge: Cambridge University Press

[B15] Scott J Social network analysis: a handbook 2000 2 London: Sage

[B16] Borgatti SP Mehra A Brass DJ Labianca G Network analysis in the social sciences Science 2009 323 892 895 10.1126/science.1165821 19213908

[B17] Newman MEJ Watts DJ Strogatz SH Random graph models of social networks Proc Natl Acad Sci U S A 2002 19 2566 2572 11875211 10.1073/pnas.012582999 PMC128577

[B18] Knoke D Burt R Burt R, Minor M Prominence Applied Network Analysis 1983 Newbury Park, CA: Sage 195 222

[B19] Valente TW Pumpuang P Identifying opinion leaders to promote behavior change Health Educ Behav 2007 34 881 896 17602096 10.1177/1090198106297855

[B20] Braithwaite J An empirical assessment of social structural and cultural change in clinical directorates Health Care Analysis 2006 14 185 193 10.1007/s10728-006-0025-5 17214253

[B21] Braithwaite J Westbrook M Rethinking clinical organisational structures: an attitude survey of doctors, nurses and allied health staff in clinical directorates J Health Serv Res Policy 2005 10 10 17 10.1258/1355819052801778 15667699

[B22] Braithwaite J Between group behaviour in health care: gaps, edges, boundaries, disconnections, weak ties, spaces and holes. A systematic review BMC Health Serv Res 2010 10 330 10.1186/1472-6963-10-330 21134295 PMC3004899

[B23] Marsden P Marsden P, Lin N Brokerage behaviour in restricted exchange networks Social structure and network analysis 1982 Beverley Hills: Sage 201 218

[B24] Burt RS Brokerage and closure: an introduction to social capital 2005 New York: Oxford University Press

[B25] Marrone JA Tesluk PE Carson JB A multilevel investigation of antecedents and consequences of team member boundary-spanning behavior Acad Manag Ann 2007 50 1423 1439 10.5465/AMJ.2007.28225967

[B26] Cummings J Cross R Structural properties of work groups and their consequences for performance Social Networks 2003 25 197 210 10.1016/S0378-8733(02)00049-7

[B27] Cross R Parker A The hidden power of social networks: understanding how work really gets done in organizations 2004 Boston, Massachusetts: Harvard Business School Press

[B28] Krackhardt D Hanson JR Informal networks: the company behind the charts. 1993 71 104 111 10127036

[B29] Cross R Borgatti S Parker A Making invisible work visible: using social network analysis to support strategic collaboration Calif Manage Rev 2002 44 25 46 10.2307/41166121

[B30] Tsai W Ghoshal S Social capital and value creation: the role of intrafirm networks Acad Manage J 1998 41 464 476 10.2307/257085

[B31] Granovetter M The strength of weak ties: a network theory revisited 1982 Beverly Hills, CA: Sage

[B32] Chambers D Wilson P Thompson C Harden M Social network analysis in healthcare settings: a systematic scoping review PLoS ONE 2012 7 e41911 10.1371/journal.pone.0041911 22870261 PMC3411695

[B33] Jansson SM Benoit C Casey L Phillips R Burns D In for the long haul: knowledge translation between academic and nonprofit organizations 2010 20 131 143 10.1177/1049732309349808 PMC4441620 19801416

[B34] Tetroe JM Graham ID Foy R Robinson N Eccles MP Wensing M Health research funding agencies’ support and promotion of knowledge translation: an international study Milbank Q 2008 86 125 155 10.1111/j.1468-0009.2007.00515.x 18307479 PMC2690338

[B35] Field A Baxter K Terry SF From bench to practice to population health impact: barriers to realizing the public health and clinical promise of basic scientific discovery Genet Test Mol Biomarkers 2011 15 191 192 10.1089/gtmb.2011.1518 21428744

[B36] Tageja N Bridging the translation gap - new hopes, new challenges Fundam Clin Pharmacol 2011 25 163 171 10.1111/j.1472-8206.2010.00903.x 21155875

[B37] Tenenbaum JD Whetzel PL Anderson K Borromeo CD Dinov ID Gabriel D Kirschner B Mirel B Morris T Noy N The Biomedical Resource Ontology (BRO) to enable resource discovery in clinical and translational research J Biomed Inf 2011 44 137 145 10.1016/j.jbi.2010.10.003 PMC3050430 20955817

[B38] Zierhut H Austin J How inclusion of genetic counselors on the research team can benefit translational science Sci Transl Med 2011 3 74cm77 10.1126/scitranslmed.3001898 PMC3753290 21411737

[B39] Woolf SH The meaning of translational research and why it matters JAMA 2008 299 211 213 10.1001/jama.2007.26 18182604

[B40] Zerhouni EA Translational and clinical science: time for a new vision N Engl J Med 2005 353 1621 1623 10.1056/NEJMsb053723 16221788

[B41] National Institutes of Health Clinical and Translational Science Awards 2013 http://www.ncats.nih.gov/research/cts/ctsa/ctsa.html, accessed 2 Sept 2013

[B42] Goldblatt EM Lee W-H From bench to bedside: the growing use of translational research in cancer medicine Am J Transl Res 2010 2 1 18 20182579 PMC2826819

[B43] Rowley E Morriss R Currie G Schneider J Research into practice: collaboration for leadership in applied health research and care (CLAHRC) for Nottinghamshire, Derbyshire, Lincolnshire (NDL) Implement Sci 2012 7 40 10.1186/1748-5908-7-40 22553966 PMC3441357

[B44] Bartlett RH Translating innovation: Beethoven, Gross, Krummel, and Georgeson J Pediatr Surg 2011 46 18 21 10.1016/j.jpedsurg.2010.09.057 21238634

[B45] Bergman D Beck A Moving from research to large-scale change in child health care Acad Pediatr 2011 11 360 368 10.1016/j.acap.2011.06.004 21783449

[B46] Rangachari P Knowledge sharing networks related to hospital quality measurement and reporting Health Care Manage Rev 2008 33 253 263 10.1097/01.HMR.0000324910.26896.91 18580305

[B47] Burt RS Structural holes and good ideas Am J Sociol 2004 110 349 399 10.1086/421787

[B48] Obstfeld D Social networks, the *tertius iungens* orientation, and involvement in innovation Adm Sci Q 2005 50 100 130

[B49] Battilana J Casciaro T Change agents, networks, and institutions: a contingency theory of organizational change Acad Manag J 2012 55 381 398 10.5465/amj.2009.0891

[B50] Haines V Godley J Hawe P Understanding interdisciplinary collaborations as social networks Am J Community Psychol 2011 47 1 11 10.1007/s10464-010-9374-1 21063766

[B51] Tetroe JM Graham ID Foy R Robinson N Eccles MP Wensing M Durieux P Légaré F Nielson CP Adily A Health research funding agencies’ support and promotion of knowledge translation: an international study Milbank Quarterly 2008 86 125 155 10.1111/j.1468-0009.2007.00515.x 18307479 PMC2690338

[B52] Wasserman S Faust K Social network analysis: Methods and applications 1994 Cambridge university press

[B53] Westfall JM Mold J Fagnan L Practice-based research - “blue highways” on the NIH roadmap JAMA 2007 297 403 406 10.1001/jama.297.4.403 17244837

[B54] Long JC Cunningham FC Braithwaite J Network structure and the role of key players in a translational cancer research network: a study protocol BMJ Open 2012 2 10.1136/bmjopen-2012-001434 PMC3383981 22734122

[B55] Friedkin NE The development of structure in random networks: an analysis of the effects of increasing network density on five measures of structure Social Networks 1981 3 41 52 10.1016/0378-8733(81)90004-6

[B56] Dauphinée D Martin JB Breaking down the walls: thoughts on the scholarship of integration Acad Med 2000 75 881 886 10.1097/00001888-200009000-00008 10995608

[B57] Brass DJ Being in the right place: a structural analysis of individual influence in an organization Adm Sci Q 1984 29 518 539 10.2307/2392937

[B58] Borgatti SP Everett MG Freeman LC UCInet for Windows: software for social network analysis 2002 6 Harvard: Analytic Technologies

[B59] Stokols D Misra S Moser RP Hall KL Taylor BK The ecology of team science: understanding contextual influences on transdisciplinary collaboration Am J Prev Med 2008 35 S96 S115 10.1016/j.amepre.2008.05.003 18619410

[B60] Goodwin N Perri G Peck P Freeman T Posaner R Managing across diverse networks: lessons from other sectors. Report to the national coordinating centre for the NHS service delivery and organisation R&D programme 2004 London: London School of Hygiene and Tropical Medicine

