# Supplementary material for: Who are the key players in a new translational research network?
Source: BMC Health Serv Res. 2013 Aug 30;13:338. doi: 10.1186/1472-6963-13-338 (PMC3844428; doi:10.1186/1472-6963-13-338)
Supplement: Additional file 1 — Ethics Approvals. [file 1472-6963-13-338-S1.pdf]

Ethics approvals

This project is part of the ARC Discovery Project DP0986493: “Evaluating communities of practice and social-professional networks: The development, design, testing, refinement, simulation and application of an evaluation framework. “

Ethics approvals were sought and obtained from the following organisations:

- University of New South Wales (HREC reference no: 09085)
- South Eastern Sydney Northern Sector Local Health Network (HREC reference no: 11/152 LNR/11/POWH/254)
- Calvary Health Care Sydney (HREC reference no: 11/G/164).
