# Supplementary material for: Who are the key players in a new translational research network?
Source: BMC Health Serv Res. 2013 Aug 30;13:338. doi: 10.1186/1472-6963-13-338 (PMC3844428; doi:10.1186/1472-6963-13-338)
Supplement: Additional file 2 — TRN Collaboration Survey 1 for BMC HSR. [file 1472-6963-13-338-S2.pdf]

UNSW

Translational Research Network Collaboration Survey

Abridged version of the on-line survey
prepared for inclusion with BMC Health Services Research article
"Who are the key players in a new translational research network?"

Never Stand Still | Faculty of Medicine | **School of Public Health and Community Medicine**

Welcome

Thank you for taking part in the TRN (translational research network) Collaboration Survey. The survey will help us to understand current patterns of collaboration and communication within the network and how we can optimize the network's efficiency.

Social network surveys map the relationships between participants by surveying all members and then combining their answers. We need to use members' names on the survey in order to construct each person's web of contacts however once submitted, data will be de-identified. Individuals and institutions will be anonymous in all reporting of results.

The survey will take 20 minutes to complete. Part 1 asks you about your work and contacts outside of the TRN, your role in the TRN so far and your views on how it works.

Part 2 asks you to map your TRN social network by considering different types of links to other TRN members.

1. I agree to participate in this survey.
   - ☐ Yes
   - ☐ No
2. Where do you currently work? (Please select all that apply)
   - ☐ Hospital #1
   - ☐ Hospital #2
   - ☐ Hospital #2 Clinical School
   - ☐ Hospital #3
   - ☐ Hospital #4
   - ☐ Hospital #5
   - ☐ Hospital #6
   - ☐ Health District A
   - ☐ Pathology Service #1
   - ☐ Research Centre
   - ☐ University A
   - ☐ University B
   - ☐ University C
   - ☐ University D
   - ☐ University E
   - ☐ University F
   - ☐ Other (please specify):

3. Have you ever worked in any of these institutions previously? (Please select all that apply)
   - ☐ Hospital #1
   - ☐ Hospital #2 …..

- ☐
- ☐ None of the above
- ☐ Other (please specify):

3. Which of the following makes up the **major** component of your work? (Please select one)
   - ☐ Direct patient care
   - ☐ Administration or management
   - ☐ Research
   - ☐ Laboratory work
   - ☐ Teaching / supervision of students or junior staff
   - ☐ Other (please specify: ....)
4. What other activities form a part of your work? (Please select all that apply)
   - ☐ Direct patient care
   - ☐ Administration or management
   - ☐ Research
   - ☐ Laboratory work
   - ☐ Teaching / supervision of students or junior staff
   - ☐ Other (please specify: ....)
5. Which of the following titles would most closely describe you? (Please select one)
   - ☐ Clinician
   - ☐ Manager
   - ☐ Researcher
   - ☐ Academic
   - ☐ Clinician-researcher
   - ☐ Other (please specify: ....)
6. Do your colleagues consider you an expert in your specialty area?
   - ☐ Yes
   - ☐ No
7. If yes, what is that area of expertise: ....
8. How many years have you been in working in **this field**?
   - ☐ < 1 year
   - ☐ 1-5 years
   - ☐ 6-10 years
   - ☐ 11-20 years
   - ☐ > 20 years
9. Do you have more than one qualification that is relevant to TRN? (E.g. pathologist and registered nurse).
   - ☐ Yes (please specify)
   - ☐ No
10. Have you been involved in any research **in the past** that you would describe as translational, irrespective of its outcome?
    - ☐ Yes
    - ☐ No
11. The following is a list of things that TRN members have named as being important in **facilitating** the TRN's aims of translating research into clinical practice. On a scale of 1 to 5,

how important do you think each factor is, where 1 is very unimportant, 2 is unimportant, 3 neutral, 4 important and 5 is very important?

a) Strong leadership and direction
b) Regular communication (e.g. newsletters, emails) from the TRN
c) Support from Project Officers and / or Translational Research Fellows
d) Access to shared resources, e.g. databases
e) Access to expertise
f) Projects are focused on patient outcomes
g) The connections that people in the TRN have already with external people and resources
h) Adequate funding
i) The support of my organisation for TRN activities

12. The following is a list of things that TRN members have named as being important **barriers** to the TRN's aims of translating research into clinical practice. On a scale of 1 to 5, how significant a barrier do you think each factor is, where 1 is not significant at all, 2 minimally significant, 3 is neutral, 4 is significant and 5 is very significant?
    a) Lack of time for TRN activities on top of one's usual work
    b) Lack of interest from my colleagues or potential collaborators
    c) Lack of support in the clinical setting
    d) Mismatched expectations of scientists / researchers or clinicians
    e) Poor communication (e.g. Governing Body not listening to members, too many emails)
    f) Lack of adequate funding
    g) Lack of incentives for either scientists / researchers or clinicians
    h) Poor or absent connections between scientists / researchers and clinicians
    i) Difficulties in managing collaborations
    j) Too much work to do to satisfy the funding body (e.g. reporting)
13. Are you a member of another relevant network, group or forum (e.g. ACI clinical network, St Elsewhere Nurses' group; online physicians' forum)?
    ☐ Yes (name of group /s ……………)
    ☐ No
14. Considering your involvement with the TRN, which of the following statements are true?
    a. **Method** of joining the TRN (Please select all that apply)
       ☐ I was involved in writing the initial TRN proposal
       ☐ I was invited to join the TRN by another member
       ☐ I joined the TRN myself without an invitation from another member
       ☐ I was required to join by a superior (not a member)
       ☐ Other (please specify):
    b. **Reasons** for joining (Please select all that apply)
       ☐ I joined because I am deeply committed to the TRN's aims and objectives
       ☐ I joined for the networking and career opportunities the TRN may provide
       ☐ I'm an expert in my field and I want to offer my expert advice
       ☐ I joined so I would have access to research support and expertise
       ☐ I joined so I could get funding for a project
       ☐ I just wanted to be involved

☐ I was required to join by a superior
☐ Other

c. What form of communication have you had with TRN members so far? (Please select all that apply)

☐ I have received a group email from TRN
☐ I have responded to a group email from TRN
☐ I have received the TRN Newsletter
☐ I have read the TRN Newsletter
☐ I have initiated a personal email or phone call to another member concerning TRN
☐ I have responded to a personal email or phone call from another member concerning TRN
☐ None of the above

15. What other activities have you participated in with other members of the TRN: (Please select all that apply):

☐ I have attended a formal meeting about the TRN
☐ I have taken part in an informal meeting or discussion about TRN
☐ I have initiated an informal meeting or discussion about the TRN
☐ I have circulated the TRN newsletter to colleagues
☐ I am involved in a TRN projec**t**
☐ I'm considering with whom I can collaborate on a project but haven't approached anyone yet
☐ I have provided advice for a TRN project being run by others
☐ I am considering ways the TRN can disseminate their findings more widely
☐ Other

In the next section we are going to ask you to define your relationship with other members of the TRN. Below is a list of full TRN members *(example only shown here).*

Jane Doe, Physician, Hospital #1
John Smith, research fellow, University A

16. Firstly, please select your own name from the list.
17. Select from the member list anyone with whom you have consulted or collaborated regarding any **TRN research project**. If no-one fits this category please check "none of the above."
18. Select from the member list anyone with whom you have collaborated regarding **dissemination of TRN objectives or findings**. If no-one fits this category please check "none of the above."
19. Please indicate the people on the member list that you think are the **most powerful** in terms of setting the TRN agenda or direction. You may select yourself. If no-one fits this category please check "none of the above."
20. Please indicate the people on the member list that you think will be the **most influential** in terms of achieving the outcome of changing clinical practices. You may select yourself. If no-one fits this category please check "none of the above."

21. Networks are about making new contacts and linking people together. Please indicate on the member list people you would describe as **highly connected** members. You may select yourself. If no-one fits this category please check “none of the above.”

That is the end of the survey. Thank you for your time.
